# A Cross-Sectional Analysis of Parental Behavior and Adolescent Mental Health in Mexico: Insights into Excessive Alcohol Intake, Tobacco Use, Suicidal Behavior, and Depressive Symptomatology

**DOI:** 10.3390/healthcare12060641

**Published:** 2024-03-13

**Authors:** Luz Myriam Reynales-Shigematsu, Leonor Rivera-Rivera, Marina Séris-Martínez, Belen Saenz-de-Miera

**Affiliations:** 1 Population Health Research Center, National Institute of Public Health, Cuernavaca 62100, Mexico; lreynales@insp.mx (L.M.R.-S.); lrivera@insp.mx (L.R.-R.); 2 Department of Economics, Autonomous University of Baja California Sur, La Paz 23085, Mexico; b.saenzdm@uabcs.mx

**Keywords:** adolescent, family, intergenerational, smoking, alcohol drinking, suicidal behavior, child sexual abuse, depressive symptomatology

## Abstract

Depression, suicidal behavior, excessive alcohol intake, and tobacco use are the main mental health problems in adolescents. To address these problems, it is necessary to understand the many factors associated with them, including parental factors. The aim of this study was to assess the associations between parental behavior and mental health problems in adolescents in Mexico. Data from the National Health and Nutrition Survey (ENSANUT) 2018–2019, representative for Mexico, were used. Households in which a parent–adolescent child pairing was identified (regardless of family type) were selected; n = 8758 households. The four outcomes of interest that were measured in the adolescents were: excessive alcohol intake, tobacco use, suicidal behavior, and depressive symptomatology. Logistic regression models using the adjusted odds ratio (AOR) and 95% confidence interval (95% CI) were estimated. Adolescents whose parents used alcohol or tobacco and reported depressive symptoms and suicidal behavior were more likely to present these behaviors themselves (AOR = 1.47, 95% CI: 1.17–1.85; AOR = 2.26, 95% CI: 1.51–3.39; AOR = 2.61, 95% CI: 1.88–3.61; AOR = 1.74, 95% CI: 1.16–2.61, respectively). Child sexual abuse was also strongly associated with the four outcomes of interest in adolescents (AOR = 1.89, 95% CI: 1.06–3.36 for excessive alcohol intake; AOR = 2.97, 95% CI: 1.49–5.91 for tobacco use; AOR = 5.15, 95% CI: 3.27–8.09 for depressive symptoms; AOR = 6.71, 95% CI: 4.25–10.59 for suicidal behavior). The family constitutes the central nucleus of care for children and adolescents; therefore, any effort to promote adolescent mental health must necessarily involve their parents and family.

## 1. Introduction

Worldwide, 14% of the adolescent population experiences some mental health problem, which poses a major public health challenge [1]. Depression [2], suicidal behavior [3], alcohol intake [4], and tobacco use [5] are the main mental health problems in adolescents. In 2019, the prevalence of depression in the global population aged 15–19 years was 3.8% [6], while suicidal ideation and suicide attempts reached 18% and 6%, respectively [7]. At the same time, this population accounts for a quarter of alcohol consumption worldwide, and 12% of adolescents between 13 and 15 years old use tobacco [4,5].

In Mexico, mental health problems in adolescents are also a persistent public health concern. Recent national surveys indicate that 7.1% of the population between ages 10 and 19 present at least two depressive symptoms [8], while suicidal ideation and suicidal attempts are experienced by 7.6% and 6.5%, respectively [9]. In addition, around two out of ten (or 20.6%) Mexican adolescents report alcohol intake, 13.9% report excessive alcohol intake [10], and 4.6% use tobacco [11].

To address mental health problems more effectively in the adolescent population, it is necessary to understand the many factors associated with these problems. In the ecological model, family [12], parental, and individual factors [13] stand out. In particular, it has been shown that family, as one of the pillars of society, depending on its structure and socioeconomic conditions [14,15], can influence mental health and substance use in children [15,16]. Parental attitudes and behaviors have been associated with certain mental health problems that are observable during childhood, adolescence, and even adulthood [17]. Specifically, harmful or problem drinking by parents or caregivers in the household can disrupt family relationships and lead adolescents to chronic stress, injury, and substance abuse [18]. Likewise, tobacco and other substance abuse by parents can create high stress levels in children and lead to increased substance use [19,20], depression, and suicidal behavior [21], especially in adolescence. It has been shown that offspring of depressed parents are also more likely to develop depression themselves [22]. Although we currently know that suicidal behavior is multifactorial, previous studies have reported that exposure to parental suicide has been associated with an increased risk for suicide and suicide attempts in offspring [23].

Regarding individual factors, these health problems affect males and females differently: the latter are more likely to develop depression, while the former are more susceptible to substance use [24]. Additionally, as an individual factor, a history of child sexual abuse (CSA) is associated with substance use in adolescents as a coping mechanism [25].

This topic is a relevant public health problem in Mexico and worldwide; it affects the mental health of future generations and represents an important burden among young people [26]. In 2015, Mexico joined the 2030 Agenda for Sustainable Development, in which Goal 3 is to “ensure a healthy life and promote wellbeing for all at all ages”, which includes promoting mental health, as well as strengthening the prevention and treatment of addictive substance abuse in adolescents [27]. However, programs for addictions, depression, and suicidal behaviors have been implemented in an isolated manner, hindering the country’s progress toward this goal. It was not until recently (2020) that the Specific Plan for Mental Health and Addictions was created [28] with the aim of inserting the prevention and treatment of addictions into a community model of mental health, but, in practice, the coordinated prevention strategy and universal access to mental health care is not yet guaranteed.

The present study aims to assess the associations between parental behavior and mental health problems in adolescents in Mexico. The hypothesis is that adolescents who have parents with depressive symptoms, suicidal behavior, and consume alcohol and/or tobacco are more likely to be at risk of having the same behaviors. The findings of this study could be useful for policymakers to design and implement appropriate strategies to promote mental health in Mexican adolescents and worldwide.

## 2. Materials and Methods

### 2.1. Data

The data for this study came from the National Health and Nutrition Survey 2018–2019 (Encuesta Nacional de Salud y Nutrición, ENSANUT), a face-to-face household survey with national (and state) representativity. The ENSANUT survey is conducted every five years and has the objective of assessing the health status of different age groups, including adolescents (10 to 19 years old) and adults (20 years old or more), in Mexico. These surveys are publicly available; their databases and questionnaires have been used for research purposes and statistical analysis to generate information that can support healthcare decision-making.

The ENSANUT uses probabilistic, multistage sampling procedures to select households [29]. First, a household questionnaire is given to an informant aged 18 years or older (head of household, spouse, or other household member) who lives in the household and knows the information regarding all the household members. Then, whenever possible, one person from each age group within the household is selected to answer a questionnaire specific to their age group. The survey was approved by the Ethics, Research, and Biosafety Commissions of the National Institute of Public Health.

To analyze family associations between parents and children, we restricted the sample to households where an adult and an adolescent were identified, resulting in n = 13,999. We then restricted the sample to households where the parent–child binomial was identified regardless of family type, i.e., single-parent, nuclear, or other (n = 11,570). Finally, households with adolescents who were married or living together as a couple were excluded for a final analytical sample of 8758 households.

### 2.2. Measures

#### 2.2.1. Dependent Variables

The four outcomes of interest were excessive alcohol intake, tobacco use, suicidal behavior, and depressive symptomatology in adolescents 10 to 19 years old.

*Excessive alcohol intake* was defined as 5 or more drinks at a time for males, and 4 or more at a time for females, where one drink is equivalent to a bottle, can, or glass of beer; a cocktail or mix; or a shot of tequila or mezcal. Specifically, a dichotomous excessive alcohol intake variable was created with response options of 0 = never having drunk alcohol excessively, and 1 = drinking alcohol excessively (either daily, weekly, monthly, or occasionally) [30].

*Tobacco use*. The ENSANUT uses standardized questions from global tobacco surveys. Thus, the variable Current smoker was used, built through the question: *Do you currently smoke?* As per the international standard definition, those who reported currently smoking, either daily or occasionally, were considered current smokers [31].

*Suicidal behavior.* This variable was defined using information of suicidal ideation (ever thought about committing suicide) and suicide attempt (ever hurt yourself with the purpose of taking your own life). Specifically, a dichotomous variable was defined with a response option of 1 if at least one of the aforementioned behaviors was present, and 0 otherwise [32].

*Depressive symptomatology*. This variable was defined using the CESD-7, the abbreviated version of the Depression Scale of the Center for Epidemiologic Studies, which has been previously used with the ENSANUT (Cronbach’s alpha = 0.83) and measures the frequency of depressive symptoms (0–21 points) experienced during the week prior to the survey. The presence of depressive symptoms was used as a cutoff value, i.e., 9 or more points on the CESD-7 [33]. For our dataset, Cronbach’s alpha was calculated (Cronbach’s alpha = 0.73).

#### 2.2.2. Independent Variables

- Family factors

*Socioeconomic level (SEL).* An index was created using principal component analysis, based on information on the characteristics of dwellings and household assets [34]. For the analysis, tertiles of SEL (low, medium, and high) were defined.

*Family type.* Three types of families were considered: (1) nuclear (father, mother, and teenager); (2) single-parent (father or mother, and teenager); (3) other family types (any combination other than the previous two). This last category was taken as the reference.

- Parental factors

*Excessive alcohol intake in the past month.* In the case of parents, excessive alcohol intake refers to alcohol intake of 5 or more drinks at a time for fathers and 4 or more drinks for mothers in the past 30 days. The variable was dichotomized into 1 = excessive alcohol intake at least once in the past 30 days, and 0 = otherwise [30].

*Current smoker.* This variable was defined from the same question used for adolescents, in line with the standard international definition: those who reported smoking cigarettes daily, weekly, monthly, or occasionally were considered current smokers [31].

*Depressive symptomatology*. Defined the same as for adolescents, based on the abbreviated version of the CESD-7, with a cutoff point of ≥9 to consider the presence of depressive symptomatology [33].

*Suicidal behavior.* This variable also followed the definition for adolescents, i.e., =1 if the father/mother reported suicidal ideation or suicide attempts, and =0 otherwise [32].

- Individual factors

*Childhood sexual abuse (CSA)*. Measured by the question, *Throughout your life, did someone grope you, touch, or stroke any part of your body, or have sexual relations with you when you were little?*, with response options “(1) Yes, before age 12”, “(2) Yes, when I was 12 or older”, “(3) No, never”, “(4) No response”, “(5) Does not know/Does not remember”. Responses were coded as No = 0, Yes = 1 [32].

*Gender* (male/female) and *age* (10–13 years, 14–17 years, 18–19 years) were also included as individual controls.

### 2.3. Statistical Analysis

The necessary weighting for complex survey designs was used to conduct descriptive, bivariate, and multivariate analyses. First, the prevalence of each outcome of interest was estimated and standard statistical tests (chi-squared) were obtained to assess differences between comparison groups (categories of independent variables). Second, to assess the association between parental factors (main independent variables) and mental health problems in adolescents (dependent variables), multivariate logistic regression models were estimated, controlling for conceptual and statistically relevant variables included in Table 1, to obtain adjusted odds ratios (AOR) with 95% confidence intervals (95% CI); age was included as a continuous variable. For all the statistical analysis, the software Stata 17 was used [35].

## 3. Results

### 3.1. Sample Description

The households in the sample were equally distributed among the three socio-economic levels (low, middle, and high, with approximately 33% each), and almost 70% were nuclear families, i.e., families with both parents and at least one adolescent child. About 20% of the parents reported excessive alcohol intake, 15.3% were current smokers, 15% had depressive symptomatology, and 6.5% suicidal behavior. Just over half of the adolescents (54.2%) were between 14 and 17 years old, 52.6% were male, 18% reported excessive alcohol intake, while 6.1% were current smokers. In addition, 6.4% of adolescents presented depressive symptomatology and 7.0% suicidal behavior; 2.5% had a history of CSA (Table 1).

### 3.2. Prevalence of Mental Health Problems in Adolescents

No clear socioeconomic gradient in the prevalence of mental health problems in adolescents was observed, although the prevalence of excessive alcohol intake was higher at the medium and high SELs (19.7% and 18.7%, respectively, vs. 15.0% at the low level; *p* value = 0.004), while the prevalence of smoking was higher at the medium and low SELs (8.0% and 6.2%, respectively, vs. 4.0% at the high level; *p* value < 0.001); depressive symptomatology and suicidal behaviors were similar across all SELs (Table 2). Adolescents from nuclear families had the lowest prevalence of excessive alcohol intake (*p* value = 0.008) and tobacco use (*p* value < 0.001) compared to other types of families. 

In general, the prevalence of mental health problems in adolescents was related to excessive alcohol intake and tobacco use in parents (Table 2). Specifically, the prevalence of excessive alcohol intake was higher in adolescents whose parents reported having consumed alcohol excessively in the past month (*p* value = 0.001). In addition, the prevalence of excessive alcohol intake and tobacco use was higher in adolescents whose parents were current tobacco users, had depressive symptomatology, or a history of suicidal behavior (Table 2). Adolescents whose parents had a history of depressive symptomatology or suicidal behavior had themselves a higher prevalence of depressive symptomatology and suicidal behavior (Table 2).

As for individual level factors, male adolescents compared to females had a higher prevalence of excessive alcohol intake and tobacco use (Table 2). Female adolescents, however, presented a higher prevalence of depressive symptomatology and suicidal behavior (*p* value < 0.001). The highest prevalence of excessive alcohol intake, smoking, depressive symptomatology, and suicidal behavior was observed in the 18–19 age group. Importantly, the prevalence of each of the four outcomes in adolescents was higher among those with CSA (Table 2).

### 3.3. Factors Associated with Mental Health Problems in Adolescents

Logistic regression models indicated that, relative to adolescents with a low SEL, adolescents with a middle SEL had higher odds of excessive alcohol intake (AOR = 1.31; 95% CI: 1.03–1.66), whereas adolescents with a high SEL had lower odds of using tobacco (AOR = 0.50; 95% CI: 0.35–0.72) (Table 3). Adolescents from nuclear families had also lower odds of using tobacco (AOR = 0.64; 95% CI: 0.41–0.98) compared to other family types, but family type was not associated with excessive alcohol intake, depressive symptomatology, or suicidal behavior in adolescents in adjusted models.

As for parental factors, it was found that adolescents whose parents reported excessive alcohol intake had 1.5 times the odds of presenting excessive alcohol intake themselves (AOR = 1.47; 95% CI: 1.17–1.85). This was also the case for tobacco use; parental smoking and adolescent smoking were strongly associated (AOR = 2.26; 95% CI: 1.51–3.39). Likewise, adolescents whose parents reported depressive symptoms had more than twice the odds of having these symptoms and 1.7 times the odds of presenting suicidal behavior themselves (AOR = 2.61; 95% CI: 1.88–3.61 and AOR = 1.71; 95% CI: 1.25–2.35, respectively). Finally, parental suicidal behavior was associated with excessive alcohol intake and suicidal behavior in adolescents (AOR = 1.77; 95% CI: 1.06–2.96 and AOR = 1.74; 95% CI: 1.16–2.61, respectively).

All adolescent individual factors considered were associated with one or more mental health problems. Specifically, each additional year of age increased the odds of excessive alcohol intake and tobacco use (AOR = 1.83; 95% CI: 1.74–1.94 and AOR = 1.72 = 1.59–1.87). In addition, females had lower odds of excessive alcohol intake and tobacco use than males (AOR = 0.51; 95% CI: 0.42–0.61 and AOR = 0.20; 95% CI: 0.14–0.31, respectively), but higher odds of presenting depressive symptoms and suicidal behavior (AOR = 1.88; 95% CI: 1.44–2.47 and AOR = 2.15; 95% CI: 1.68–2.74, respectively). A history of CSA significantly increased the odds of presenting all the outcomes of interest; in the case of depressive symptoms and suicidal behavior, the odds reached 5.2 and 6.7, respectively.

## 4. Discussion

The present analysis, based on a representative survey for Mexico, found that parental factors such as excessive alcohol intake, tobacco use, depressive symptomatology, and suicidal behavior were associated with mental health problems in adolescents. Family factors, namely SEL and type of family, were only associated with substance use in adolescents. Individual factors such as CSA also showed a strong association.

Starting with family factors, we found that adolescents at the middle SEL were 31% more likely to drink alcohol excessively, compared to those with a low SEL. That is, unlike the adult population, where a positive association between SEL and alcohol intake has been found [36], the socioeconomic gradient in adolescents is less clear [37]. Studies for other countries, such as the one by Melotti et al. for England, also found a positive association between high SEL and alcohol intake in adolescents [14]. However, a study by Bosque et al., which used data from six European countries, found that alcohol intake was only positively associated with each adolescent’s own SEL (based on weekly money received and academic development) but not so with parental SEL [38]. Another relevant finding regarding the role of SEL is that adolescents of high SEL were less likely to use tobacco. This result agrees with previous studies [15,39]. In particular, a study for Mexico found that adolescents of low SEL were more likely to use tobacco than high SEL adolescents [15]. This can be explained by the influence of money on the perception of popularity in adolescents: those with low SEL use tobacco to gain acceptance from their peers [40]. The SEL can be defined in different ways [37], for example, based on household income [14], parents’ education level [38], adolescent perception of SEL [41], among others, which measure different dimensions and may thus influence the results of the relation between alcohol intake, tobacco use, and SEL. It is important that future research considers measuring SEL at the household and individual levels to clarify these associations.

We also found that adolescents living in nuclear families were less likely to use tobacco relative to other family types. These results agree with other studies, which show that family characteristics are associated with substance use in adolescents [15,16]. A variety of mechanisms have been proposed to explain the association between family structure and adolescent behavior; one of them states that a nuclear family tends to have sufficient personal and interpersonal resources for the supervision and development of close ties with adolescents, thus constituting a protective factor against adolescent substance use [16].

Both alcohol and tobacco use harm not only the parents who use them but also their children by potentially influencing their behavior toward these substances. Our results suggest that adolescents whose mother and/or father use alcohol or tobacco are more likely to use these substances themselves (AOR: 1.47, *p* value < 0.01; AOR: 2.26, *p* value < 0.001 respectively). These results have been observed in previous studies, reporting a higher risk of alcohol and tobacco intake in the children of users of these substances [15,19,42]. Various theories have tried to explain the association between parental behavior and its effect on children’s behavior. According to the social learning theory, adolescents may begin to drink alcohol and use other substances by observing and imitating their parents’ drinking patterns and associated behaviors [18]. Likewise, consistent with expectations and with the findings of other research, this study found that adolescents whose parents had depressive symptomatology had a greater likelihood of presenting such behavior (AOR: 2.61, *p* value < 0.001) [43,44]; the same association was found for suicidal behavior (AOR: 1.74 *p* value < 0.01), possibly due to social as well as biological factors and genetic vulnerability [43,45,46].

Among individual factors, our study shows that there is a greater risk of mental health problems as adolescents grow (Table 3). This reveals the need to implement interventions at an early age. Regarding differences by gender, we found that males present greater risk of substance abuse compared to females (although this effect is actually narrowing) [47], while females present a greater risk of depression and suicidal behavior, which is consistent with other studies [48,49].

Similar to a previous study carried out in Mexico, we found a positive association between a history of CSA and suicidal behavior in adolescents [32]. We also found that adolescents with a history of an event as traumatic as CSA were more likely to report substance use, which is commonly employed as a coping mechanism for such abuse [25]. This indicates that specific efforts to promote mental health and prevent substance use should be directed to this particularly at-risk population.

The present study is not without limitations. First, since it is a cross-sectional study, a causal relationship cannot be established between the independent and dependent variables. Second, unlike other studies that used individual SEL measurements for the adolescent population [36], in our study we were only able to measure household SEL. In future studies, it will be relevant to include both adolescent SEL and household SEL, as these indicators are measured differently and are not necessarily correlated [50]. Third, although the survey employed allows the consideration of different types of families (e.g., nuclear or single-parent households), it does not include data on peers and romantic partners; such data would be relevant, being associated with both risky and protective behaviors in adolescence [51]. Fourth, we only considered parental current substance use, but not intensity of use; future studies should explore potential dose-response effects.

## 5. Conclusions

This manuscript seeks to respond to the paradigm shift in the approach to mental health and addictions that the World Health Organization has proposed, especially in the perspective of prevention among vulnerable groups (adolescents), and also in the approach of its population interventions focused on individuals, families, and the community (https://www.who.int/teams/mental-health-and-substance-use/promotion-prevention, accessed on 1 February 2024).

**Figure 1:**
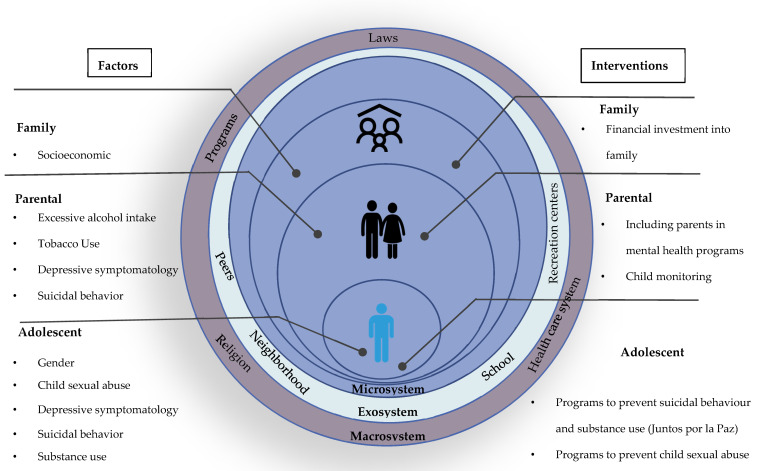
Main factors associated with adolescent mental health damage and their alignment to interventions.

Our results show that adolescents whose mother or father have mental health problems such as depression, suicidal behavior, alcohol intake, and tobacco use are more likely to be at risk to have the same behaviors. This points out the fundamental need to focus public policy efforts on adolescent mental health, considering parental and family factors. The family is the central nucleus for child and adolescent care, so any effort to promote mental health and prevent substance use must involve family-based interventions (Figure 1). In addition, special emphasis should be placed on vulnerable groups such as adolescents who have suffered abuse.

The results of this study will help strengthen government programs in adolescent health, mental health, and addictions, particularly in two emblematic programs: “Juntos por la Paz” (https://www.gob.mx/juntosporlapaz, accessed on 1 February 2024), which comprehensively addresses mental health and addiction prevention in vulnerable groups, and the “Mesa Espejo” (https://gobierno.morelos.gob.mx/noticias/morelos-implementa-la-estrategia-nacional-contra-addictions-and-suicide-together-for-it, accessed on 1 February 2024) at the state level, which carries out interventions focused on suicide prevention among adolescents, the family, and the community.

## Figures and Tables

**Table 1:** Sample characteristics of households with the parent–adolescent child pairing, Mexico 2018–2019.

Factors		N = 11,712,364 *	Percentage	95% CI
**Family factors**				
Socioeconomic level				
	Low	3,918,471	33.46	31.92–35.03
	Middle	3,867,103	33.02	31.57–34.50
	High	3,926,790	33.53	32.05–35.03
Family type				
	Other	1,566,389	13.37	12.32–14.50
	Nuclear	8,069,952	68.90	67.45–70.31
	Single-parent	2,076,023	17.73	16.64–18.86
**Parental factors**				
Sex				
	Male	4,722,089	40.32	38.83–41.82
	Female	6,990,275	59.68	58.18–61.17
Excessive alcohol intake in the past month				
	No	9,159,012	80.60	79.40–81.75
	Yes	2,204,138	19.40	18.25–20.60
Current smoker				
	No	9,905,953	84.71	83.60–85.76
	Yes	1,788,181	15.29	14.24–16.40
Depressive symptoms				
	No	9,960,092	85.04	83.93–86.08
	Yes	1,752,272	14.96	13.92–16.07
Suicidal behavior				
	No	10,947,388	93.47	92.62–94.22
	Yes	764,976	6.53	5.78–7.38
**Individual factors**				
Sex				
	Male	6,164,937	52.64	51.11–54.16
	Female	5,547,427	47.36	45.84–48.89
Age				
	10–13 years	3,300,332	28.18	26.79–29.61
	14–17 years	6,343,800	54.16	52.63–55.69
	18–19 years	2,068,232	17.66	16.58–18.79
Excessive alcohol intake				
	No	9,629,836	82.22	80.89–83.47
	Yes	2,082,528	17.78	16.53–19.11
Current smoker				
	No	10,977,972	93.94	93.10–94.68
	Yes	708,135	6.06	5.32–6.90
Depressive symptoms				
	No	10,960,957	93.58	92.85–94.25
	Yes	751,407	6.42	5.75–7.15
Suicidal behavior				
	No	10,896,358	93.03	92.27–93.73
	Yes	816,006	6.97	6.27–7.73
Sexual abuse				
	No	11,338,933	97.46	96.96–97.89
	Yes	295,107	2.54	2.11–3.04

Notes: 95% CI = 95% confidence interval. * Total weighted N was 11,712,364. Sample n was 8758 parent–adolescent child pairing.

**Table 2:** Prevalence of excessive alcohol intake, tobacco use, depressive symptomatology and suicidal behavior in adolescents 10–19 years by selected characteristics, Mexico 2018–2019.

Factors	Excessive Alcohol Intake			Tobacco Use			Depressive Symptoms			Suicidal Behavior		
	Yes	95% CI	*p* Value	Yes	95% CI	*p* Value	Yes	95% CI	*p* Value	Yes	95% CI	*p* Value
**Family factors**												
Socioeconomic level												
Low	15.00	13.22–16.97	0.004	6.20	4.99–7.69	<0.001	5.99	4.91–7.29	0.419	6.16	5.08–7.46	0.309
Middle	19.66	17.36–22.19		7.96	6.40–9.87		7.07	5.84–8.54		7.41	6.15–8.91	
High	18.71	16.72–20.86		4.04	3.21–5.07		6.19	5.18–7.38		7.33	6.20–8.65	
Family type												
Other	21.99	18.56–25.85	0.008	9.54	6.93–12.99	<0.001	6.36	4.74–8.48	0.373	8.68	6.59–11.37	0.084
Nuclear	16.65	15.13–18.29		4.85	4.04–5.81		6.17	5.39–7.04		6.44	5.67–7.32	
Single-parent	18.99	16.55–21.70		8.13	6.41–10.28		7.43	5.93–9.28		7.70	6.18–9.57	
**Parental factors**												
Excessive alcohol intake in the past month												
No	16.85	15.46–18.32	0.001	5.86	5.00–6.86	0.310	6.460	5.68–7.34	0.823	6.73	5.98–7.57	0.318
Yes	22.07	19.28–25.13		6.80	5.35–8.61		6.26	4.92–7.93		7.70	6.06–9.73	
Current smoker												
No	17.04	15.73–18.43	0.007	5.24	4.54–6.05	<0.001	6.32	5.61–7.10	0.520	6.85	6.10–7.68	0.467
Yes	21.91	18.54–25.70		10.57	7.91–13.99		6.96	5.29–9.10		7.70	5.77–10.20	
Depressive symptoms												
No	17.03	15.74–18.39	0.003	5.62	4.81–6.54	0.007	5.21	4.60–5.91	<0.001	6.16	5.45–6.96	<0.001
Yes	22.07	18.84–25.70		8.57	6.60–11.06		13.25	10.72–16.28		11.56	9.35–14.21	
Suicidal behavior												
No	16.96	15.72–18.27	<0.001	5.6	4.87–6.42	<0.001	6.10	5.43–6.83	0.001	6.51	5.83–7.27	<0.001
Yes	29.50	22.97–36.99		12.69	8.42–18.68		11.00	7.86–15.19		13.45	9.93–17.96	
**Adolescents’ individual factors**												
Sex												
Male	22.00	20.17–23.96	<0.001	9.47	8.24–10.85	<0.001	4.50	3.70–5.48	<0.001	4.51	3.77–5.40	<0.001
Female	13.09	11.65–14.68		2.26	1.61–3.18		8.54	7.48–9.73		9.69	8.57–10.94	
Age												
10–13 years	1.18	0.76–1.82	<0.001	0.44	0.19–0.99	<0.001	3.31	2.53–4.33	<0.001	5.10	3.97–6.53	0.007
14–17 years	17.24	15.54–19.07		5.58	4.58–6.80		7.27	6.31–8.36		7.66	6.73–8.70	
18–19 years	45.94	42.32–49.60		16.49	13.95–19.39		8.76	7.13–10.71		7.83	6.24–9.78	
Sexual abuse												
No	17.38	16.12–18.72	<0.001	5.77	5.03–6.61	<0.001	5.77	5.13–6.49	<0.001	6.1	5.45–6.83	<0.001
Yes	32.44	24.27–41.83		15.01	9.12–23.69		29.85	22.22–38.80		36.96	28.64–46.14	

Notes: 95% CI = 95% confidence interval.

**Table 3:** Odds of excessive alcohol intake, tobacco use, depressive symptomatology, and suicidal behavior in Mexican adolescents.

Factors	Adolescent Mental Health			
	Excessive Alcohol Intake	Tobacco Use	Depressive Symptoms	Suicidal Behavior
	AOR (95% CI)	AOR (95% CI)	AOR (95% CI)	AOR (95% CI)
**Family factors**				
Socioeconomic level				
Low	1	1	1	1
Middle	1.31 (1.03–1.66) *	1.20 (0.83–1.73)	1.14 (0.84–1.56)	1.19 (0.88–1.60)
High	1.13 (0.89–1.42)	0.50 (0.35–0.72) **	1.03 (0.77–1.39)	1.19 (0.90–1.58)
Family type				
Other	1	1	1	1
Nuclear	0.90 (0.66–1.22)	0.64 (0.41–0.98) *	1.28 (0.88–1.85)	0.88 (0.61–1.27)
Single-parent	1.00 (0.71–1.41)	1.04 (0.66–1.65)	1.18 (0.77–1.83)	0.85 (0.56–1.28)
**Parental factors**				
Excessive alcohol intake in the past month in parents				
No	1	1	1	1
Yes	1.47 (1.17–1.85) **	1.09 (0.76–1.55)	0.97 (0.70–1.33)	1.15 (0.83–1.59)
Current tobacco use in parents				
No	1	1	1	1
Yes	1.29 (0.96–1.73)	2.26 (1.51–3.39) ***	1.10 (0.79–1.56)	1.03 (0.71–1.53)
Depressive symptoms				
No	1	1	1	1
Yes	1.19 (0.93–1.52)	1.07 (0.72–1.59)	2.61 (1.88–3.61) ***	1.71 (1.25–2.35) **
Suicidal behavior in parents				
No	1	1	1	1
Yes	1.77 (1.06–2.96) *	1.58 (0.93–2.69)	1.23 (0.75–2.01)	1.74 (1.16–2.61) **
**Adolescents’ individual factors**				
Age	1.83 (1.74–1.94) ***	1.72 (1.59–1.87) ***	1.18 (1.12–1.24) ***	1.06 (1.01–1.12) *
Sex				
Male	1	1	1	1
Female	0.51 (0.42–0.61) ***	0.20 (0.14–0.31) ***	1.88 (1.44–2.47) ***	2.15 (1.68–2.74) ***
Sexual abuse				
No.	1	1	1	1
Yes	1.89 (1.06–3.36) *	2.97 (1.49–5.91) **	5.15 (3.27–8.09) ***	6.71 (4.25–10.59) ***

Notes: 95% CI = 95% confidence interval, AOR = adjusted odds ratio. All dependent variables are binary. *p* values: * < 0.05, ** < 0.01, *** < 0.001.

## Data Availability

The raw data supporting the conclusions of this article are available at https://ensanut.insp.mx/encuestas/ensanut2018/descargas.php, accessed on 1 February 2020.
